# Supplementary material for: Fire-derived phosphorus fertilization of African tropical forests
Source: Nat Commun. 2021 Aug 26;12:5129. doi: 10.1038/s41467-021-25428-3 (PMC8390740; doi:10.1038/s41467-021-25428-3)
Supplement: Supplementary file 1 — Supplementary information for fire-derived phosphorus fertilization of African tropical forests. [file 41467_2021_25428_MOESM1_ESM.pdf]

Supplementary Information for

Fire-derived phosphorus fertilization of African tropical forests

Marijn Bauters[1,2,*], Travis W. Drake[3], Sasha Wagner[4], Simon Baumgartner[1,5], Isaac Makelele[1,6], Samuel Bodé[1], Kris Verheyen[2], Hans Verbeeck[2], Corneille Ewango[7], Landry Cizungu[8], Kristof Van Oost[5], Pascal Boeckx[1]

[1] Department of Green Chemistry and Technology, Ghent University, Ghent, 9000, Belgium
[2] Department of Environment, Ghent University, Ghent, 9000, Belgium
[3] Department of Environmental Systems Science, Swiss Federal Institute of Technology, ETH Zurich, Zurich, 8092,Switzerland
[4] Department of Earth and Environmental Sciences, Rensselaer Polytechnic Institute, Troy, New York, USA
[5] Earth and Life Institute, UCLouvain, Louvain-la-Neuve, 1348, Belgium
[6] Department of Biology, Université Officielle de Bukavu, Bukavu, DRCongo
[7] Faculty of Renewable Natural Resources Management, University of Kisangani, B.P.O. 2012, Kisangani, DR Congo
[8] Soil science laboratory, Faculty of Agronomy, Université Catholique de Bukavu, Bukavu, DR Congo
* Corresponding author: Marijn.Bauters@UGent.be

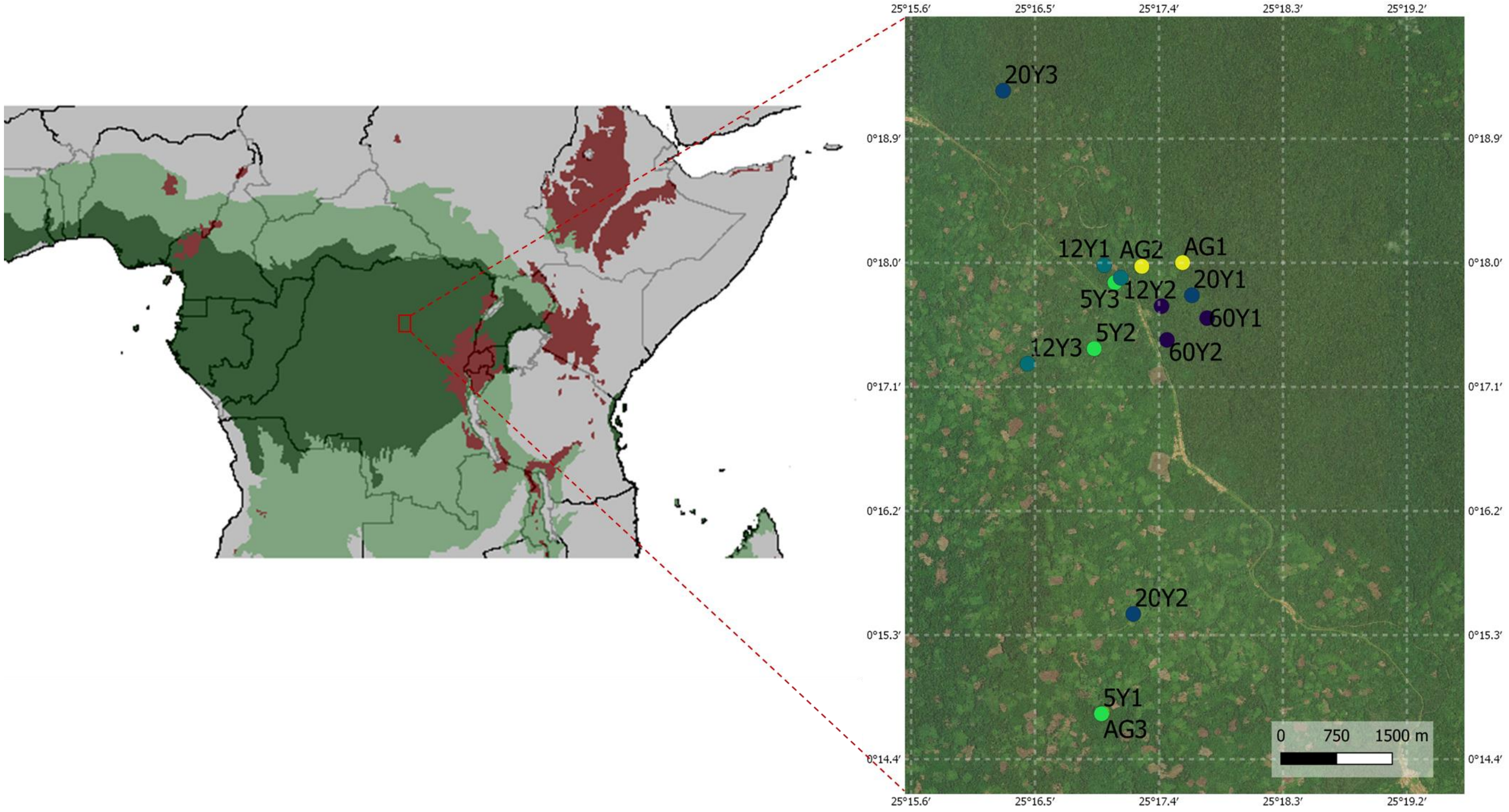

Fig S1. Overview of the study area with the triplicated experimental plots along successional stages of African forests: agricultural field (Ag), 5- (5yrs), 12- (12yrs), 20- (20yrs) and 60-year-old forest (60yrs). The coloration on the map (left) shows wet tropical forest (darkgreen), moist deciduous tropical forest (light green) and montane tropical forest (red).

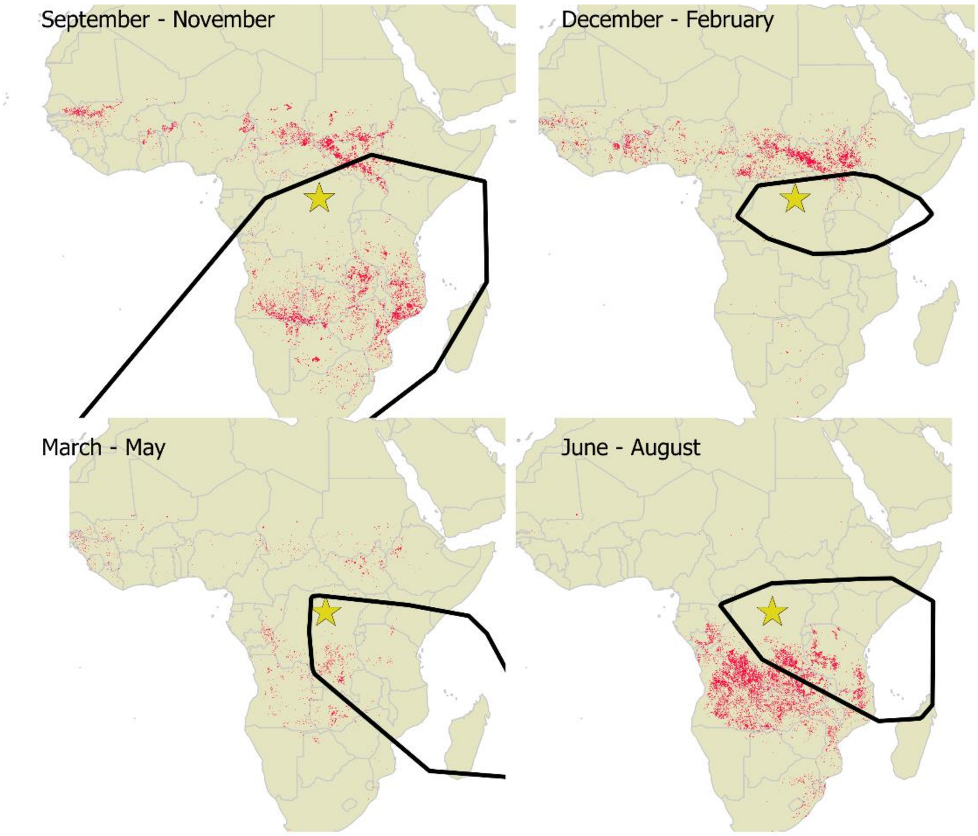

Fig S2. A visualization of the burnt area over the monitoring period (red) and a convex hull (in black) of daily wind back-trajectories (one week) arriving at the experimental site (star). This shows how winds originating from different parts of the African continent pass through burning zonings and potentially carry biomass burning-derived aerosols to the monitoring site.

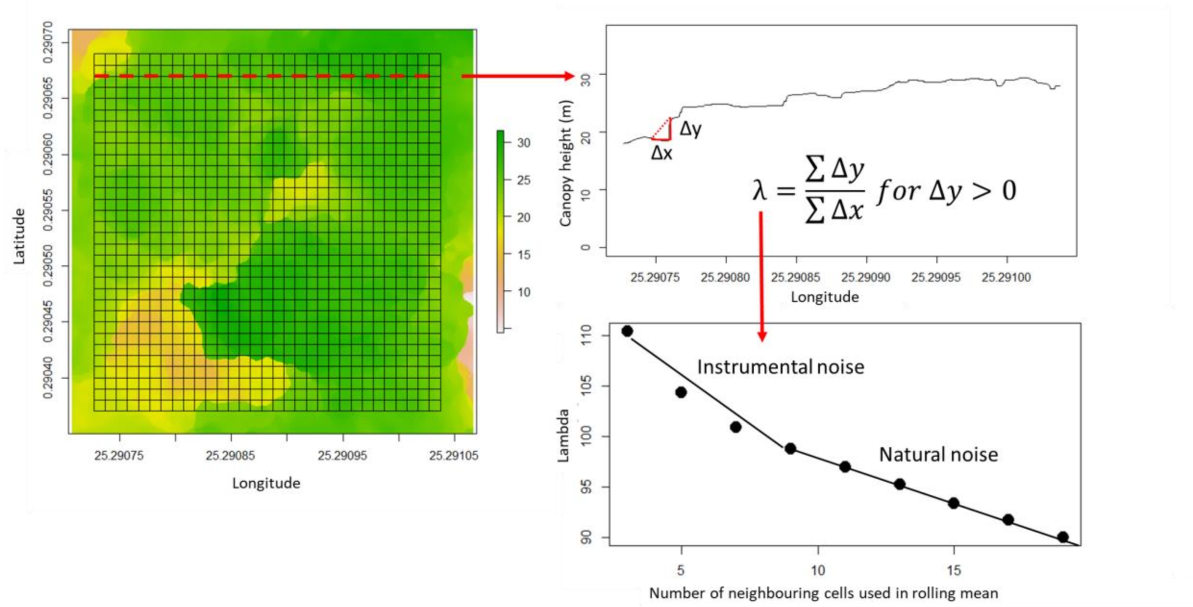

Fig S3. Overview of the lambda obstacle density parameter calculation for plot '60yr3' based on the canopy height models. Colors on the left indicate the canopy height.

Table S1. Canopy characteristics of the plots, along with canopy roughness length (Zo) and the total (TP) and dry DryP phosphorous deposition.

Plot	Forest type	Lambda	Max Tree height (m)	Zo	TP (kg P ha^{-1} yr^{-1})	DryP (kg P ha^{-1} yr^{-1})
AG1	Agriculture	0.83	0.50	0.41	1.02	0.17
AG2	Agriculture	0.61	0.50	0.30	1.55	0.38
AG3	Agriculture	0.50	0.50	0.25	1.36	0.37
5Y1	5yrs	0.53	12.33	6.58	7.14	5.77
5Y2	5yrs	0.75	12.40	9.36	3.46	1.98
5Y3	5yrs	0.61	12.40	7.52	3.74	2.48
12Y1	12yrs	0.41	15.13	6.25	4.94	3.62
12Y2	12yrs	0.33	18.47	6.04	3.45	2.41
12Y3	12yrs	0.47	21.40	10.11	4.96	3.49
20Y1	20yrs	0.50	25.47	12.79	2.70	1.48
20Y2	20yrs	0.72	21.60	15.60	3.14	1.97
20Y3	20yrs	0.92	19.60	18.09	4.02	2.77
60Y1	60yrs	0.88	27.80	24.55	6.36	5.09
60Y2	60yrs	0.88	31.67	27.72	7.57	6.32
60Y3	60yrs	0.90	28.20	25.37	14.43	12.97

Table S2. Concentrations of dissolved organic carbon (DOC), dissolved black carbon (DBC) and benzenehexacarboxylic acid (B6CA) and benzenepentacarboxylic acid (B5CA) products.

Forest Type	Sampling Date	[B6CA] µM	[B5CA] µM	[DBC] µM-C	[DBC] mg-C/L	[DOC] µM-C	[DOC] mg-C/L
12yrs	21/07/2018	0.07	0.19	14.48	0.17	482.67	5.79
20yrs	21/07/2018	0.07	0.19	14.56	0.17	318.67	3.82
Ag	21/07/2018	0.03	0.09	7.21	0.09	213.58	2.56
12yrs	28/07/2018	0.22	0.55	39.43	0.47	1570.83	18.85
20yrs	28/07/2018	0.35	0.80	57.28	0.69	1257.50	15.09
60yrs	28/07/2018	0.49	1.12	78.43	0.94	1952.50	23.43
5yrs	28/07/2018	0.23	0.55	40.04	0.48	927.50	11.13
Ag	28/07/2018	0.13	0.44	30.12	0.36	481.75	5.78
20yrs	4/08/2018	0.16	0.40	29.92	0.36	583.83	7.01
60yrs	4/08/2018	0.33	0.71	51.93	0.62	980.00	11.76
5yrs	4/08/2018	0.13	0.31	23.53	0.28	497.92	5.98
Ag	4/08/2018	0.03	0.07	5.92	0.07	121.58	1.46
12yrs	11/08/2018	0.06	0.19	14.13	0.17	503.50	6.04
20yrs	11/08/2018	0.14	0.35	26.12	0.31	528.17	6.34
60yrs	11/08/2018	0.26	0.56	41.76	0.50	971.67	11.66
5yrs	11/08/2018	0.06	0.14	11.55	0.14	360.08	4.32
12yrs	18/08/2018	0.14	0.34	25.85	0.31	639.58	7.68
20yrs	18/08/2018	0.21	0.48	35.86	0.43	744.92	8.94
60yrs	18/08/2018	0.34	0.76	55.07	0.66	1320.83	15.85
5yrs	18/08/2018	0.27	0.54	41.44	0.50	841.67	10.10
Ag	18/08/2018	0.14	0.32	24.57	0.29	342.58	4.11
12yrs	25/08/2018	0.19	0.49	35.39	0.42	1015.83	12.19
20yrs	25/08/2018	0.19	0.47	34.68	0.42	934.17	11.21
5yrs	25/08/2018	0.34	0.70	51.98	0.62	987.50	11.85
Ag	25/08/2018	0.04	0.11	9.09	0.11	222.17	2.67
12yrs	1/09/2018	0.07	0.17	13.37	0.16	285.25	3.42
60yrs	1/09/2018	0.58	0.96	74.97	0.90	1592.50	19.11
5yrs	1/09/2018	0.24	0.48	37.52	0.45	779.08	9.35
Ag	1/09/2018	0.06	0.18	13.68	0.16	317.25	3.81
12yrs	3/11/2018	0.04	0.09	7.61	0.09	199.92	2.40
20yrs	3/11/2018	0.08	0.19	15.29	0.18	281.92	3.38
60yrs	3/11/2018	0.09	0.21	16.54	0.20	356.83	4.28
5yrs	3/11/2018	0.04	0.10	7.89	0.09	179.50	2.15
12yrs	10/11/2018	0.02	0.06	5.08	0.06	106.58	1.28
20yrs	10/11/2018	0.10	0.21	17.08	0.20	497.33	5.97
60yrs	10/11/2018	0.07	0.20	15.44	0.19	932.50	11.19
5yrs	10/11/2018	0.03	0.09	7.40	0.09	264.08	3.17
Ag	10/11/2018	0.02	0.06	4.88	0.06	181.33	2.18
20yrs	17/11/2018	0.07	0.16	13.29	0.16	327.67	3.93
60yrs	17/11/2018	0.09	0.19	15.59	0.19	431.92	5.18
5yrs	17/11/2018	0.04	0.13	9.97	0.12	358.92	4.31
Ag	17/11/2018	0.07	0.19	14.54	0.17	138.17	1.66
12yrs	24/11/2018	0.06	0.15	12.38	0.15	231.00	2.77
20yrs	24/11/2018	0.08	0.19	15.16	0.18	294.08	3.53
60yrs	24/11/2018	0.08	0.17	14.61	0.18	251.08	3.01
5yrs	24/11/2018	0.05	0.11	9.21	0.11	163.67	1.96
Ag	24/11/2018	0.01	0.03	2.52	0.03	60.10	0.72
12yrs	1/12/2018	0.07	0.18	14.10	0.17	193.83	2.33
20yrs	1/12/2018	0.22	0.48	36.54	0.44	732.58	8.79
60yrs	1/12/2018	0.23	0.53	38.90	0.47	895.83	10.75
5yrs	1/12/2018	0.13	0.29	22.73	0.27	514.00	6.17
Ag	1/12/2018	0.09	0.15	13.77	0.17	379.58	4.56
12yrs	8/12/2018	0.02	0.05	4.78	0.06	106.00	1.27
60yrs	8/12/2018	0.04	0.07	6.75	0.08	144.25	1.73
5yrs	8/12/2018	0.02	0.04	3.93	0.05	76.15	0.91
Ag	8/12/2018	0.00	0.01	1.26	0.02	41.30	0.50
5yrs	22/12/2018	0.11	0.25	19.82	0.24	563.25	6.76
Ag	22/12/2018	0.02	0.04	3.42	0.04	131.33	1.58
12yrs	29/12/2018	0.17	0.38	29.40	0.35	618.08	7.42
60yrs	29/12/2018	0.18	0.41	31.48	0.38	608.08	7.30
12yrs	5/01/2019	0.07	0.15	12.60	0.15	340.75	4.09
20yrs	5/01/2019	0.15	0.35	26.26	0.32	410.08	4.92
60yrs	5/01/2019	0.11	0.23	18.63	0.22	371.50	4.46
5yrs	5/01/2019	0.04	0.12	9.33	0.11	187.92	2.26

Ag	5/01/2019	0.01	0.03	2.91	0.03	51.39	0.62
60yrs	19/01/2019	0.08	0.17	14.39	0.17	221.42	2.66
12yrs	2/02/2019	0.07	0.19	14.92	0.18	293.75	3.53
20yrs	2/02/2019	0.12	0.28	21.85	0.26	400.00	4.80
60yrs	2/02/2019	0.14	0.28	22.58	0.27	417.17	5.01
5yrs	2/02/2019	0.12	0.27	21.38	0.26	403.67	4.84
Ag	2/02/2019	0.06	0.16	12.81	0.15	266.08	3.19
12yrs	9/02/2019	0.09	0.24	18.12	0.22	314.17	3.77
12yrs	23/02/2019	0.12	0.28	21.96	0.26	478.00	5.74
Ag	23/02/2019	0.01	0.04	3.25	0.04	80.89	0.97
12yrs	2/03/2019	0.11	0.29	21.92	0.26	629.67	7.56
12yrs	9/03/2019	0.14	0.28	22.80	0.27	419.17	5.03
12yrs	23/03/2019	0.15	0.37	27.60	0.33	802.42	9.63
20yrs	23/03/2019	0.17	0.44	31.60	0.38	763.58	9.16
60yrs	23/03/2019	0.31	0.69	50.04	0.60	1609.17	19.31
5yrs	23/03/2019	0.12	0.27	21.42	0.26	464.50	5.57
Ag	23/03/2019	0.04	0.12	9.38	0.11	289.75	3.48
12yrs	30/03/2019	0.04	0.13	9.82	0.12	251.08	3.01
20yrs	30/03/2019	0.07	0.18	14.11	0.17	275.42	3.31
60yrs	30/03/2019	0.16	0.33	26.11	0.31	541.00	6.49
5yrs	30/03/2019	0.05	0.14	10.99	0.13	215.08	2.58
Ag	30/03/2019	0.01	0.03	2.78	0.03	102.50	1.23
12yrs	6/04/2019	0.01	0.03	2.82	0.03	264.08	3.17
20yrs	6/04/2019	0.07	0.20	15.31	0.18	286.58	3.44
60yrs	6/04/2019	0.18	0.37	29.53	0.35	606.17	7.27
5yrs	6/04/2019	0.05	0.14	11.07	0.13	250.75	3.01
Ag	6/04/2019	0.01	0.04	3.15	0.04	115.75	1.39
12yrs	13/04/2019	0.09	0.22	16.97	0.20	385.67	4.63
20yrs	13/04/2019	0.07	0.22	16.30	0.20	5045.00	60.54
60yrs	13/04/2019	0.27	0.56	42.23	0.51	950.83	11.41
5yrs	13/04/2019	0.08	0.21	16.01	0.19	350.83	4.21
Ag	13/04/2019	0.02	0.04	3.91	0.05	113.67	1.36
12yrs	20/04/2019	0.11	0.27	20.72	0.25	370.58	4.45
20yrs	20/04/2019	0.05	0.13	10.38	0.12	188.00	2.26
60yrs	20/04/2019	0.12	0.26	20.69	0.25	392.75	4.71
5yrs	20/04/2019	0.06	0.17	13.33	0.16	253.42	3.04
Ag	20/04/2019	0.02	0.06	4.99	0.06	118.92	1.43
12yrs	27/04/2019	0.05	0.14	10.99	0.13	222.17	2.67
20yrs	27/04/2019	0.07	0.20	15.23	0.18	273.08	3.28
60yrs	27/04/2019	0.07	0.16	13.02	0.16	267.58	3.21
5yrs	27/04/2019	0.07	0.17	13.56	0.16	233.50	2.80
Ag	27/04/2019	0.03	0.07	5.63	0.07	143.25	1.72
12yrs	4/05/2019	0.12	0.27	21.08	0.25	589.83	7.08
20yrs	4/05/2019	0.17	0.53	36.21	0.43	699.25	8.39
60yrs	4/05/2019	0.22	0.51	37.46	0.45	845.00	10.14
5yrs	4/05/2019	0.09	0.22	16.77	0.20	400.67	4.81
Ag	4/05/2019	0.01	0.03	3.10	0.04	123.58	1.48
12yrs	11/05/2019	0.04	0.09	7.40	0.09	188.08	2.26
20yrs	11/05/2019	0.04	0.10	7.89	0.09	164.83	1.98
60yrs	11/05/2019	0.13	0.25	20.73	0.25	411.75	4.94
5yrs	11/05/2019	0.04	0.11	9.11	0.11	161.58	1.94
Ag	11/05/2019	0.01	0.02	2.02	0.02	87.08	1.05
12yrs	18/05/2019	0.09	0.21	16.68	0.20	410.08	4.92
20yrs	18/05/2019	0.08	0.19	14.79	0.18	281.08	3.37
60yrs	18/05/2019	0.18	0.37	29.12	0.35	694.58	8.34
5yrs	18/05/2019	0.06	0.14	11.72	0.14	251.92	3.02
Ag	18/05/2019	0.01	0.03	3.05	0.04	91.75	1.10
12yrs	25/05/2019	0.08	0.20	15.71	0.19	373.50	4.48
20yrs	25/05/2019	0.04	0.11	9.02	0.11	207.00	2.48
60yrs	25/05/2019	0.38	1.23	77.88	0.93	1383.33	16.60
5yrs	25/05/2019	0.06	0.15	12.13	0.15	241.50	2.90
Ag	25/05/2019	0.02	0.04	3.56	0.04	115.50	1.39
12yrs	1/06/2019	0.05	0.14	10.79	0.13	292.92	3.52
20yrs	1/06/2019	0.08	0.20	15.58	0.19	307.67	3.69
60yrs	1/06/2019	0.16	0.28	24.20	0.29	560.58	6.73
5yrs	1/06/2019	0.05	0.13	10.24	0.12	228.08	2.74
Ag	1/06/2019	0.01	0.04	3.65	0.04	122.33	1.47
12yrs	8/06/2019	0.03	0.07	5.75	0.07	142.42	1.71
5yrs	8/06/2019	0.04	0.09	7.68	0.09	181.75	2.18
Ag	8/06/2019	0.00	0.01	1.16	0.01	88.00	1.06
12yrs	29/06/2019	0.12	0.30	23.00	0.28	422.50	5.07

20yrs	29/06/2019	0.14	0.34	26.02	0.31	450.33	5.40
60yrs	29/06/2019	0.16	0.35	27.02	0.32	609.00	7.31
5yrs	29/06/2019	0.12	0.29	21.79	0.26	383.25	4.60
Ag	29/06/2019	0.10	0.16	14.67	0.18	250.58	3.01
12yrs	6/07/2019	0.09	0.22	16.94	0.20	309.67	3.72
20yrs	6/07/2019	0.14	0.31	24.33	0.29	491.50	5.90
60yrs	6/07/2019	0.20	0.46	34.33	0.41	772.50	9.27
5yrs	6/07/2019	0.13	0.31	23.42	0.28	414.75	4.98
Ag	6/07/2019	0.05	0.13	10.79	0.13	220.50	2.65
12yrs	13/07/2019	0.13	0.30	23.27	0.28	440.67	5.29
20yrs	13/07/2019	0.09	0.23	18.17	0.22	317.08	3.81
60yrs	13/07/2019	0.13	0.29	22.73	0.27	459.58	5.52
5yrs	13/07/2019	0.05	0.11	9.30	0.11	178.67	2.14
Ag	13/07/2019	0.02	0.04	3.55	0.04	93.42	1.12
12yrs	20/07/2019	0.13	0.34	25.61	0.31	532.33	6.39
20yrs	20/07/2019	0.24	0.54	40.13	0.48	856.67	10.28
60yrs	20/07/2019	0.25	0.53	39.83	0.48	861.67	10.34
5yrs	20/07/2019	0.13	0.27	21.79	0.26	539.50	6.47
Ag	20/07/2019	0.06	0.13	10.51	0.13	336.25	4.04
12yrs	27/07/2019	0.13	0.40	28.37	0.34	605.67	7.27
20yrs	27/07/2019	0.13	0.30	23.27	0.28	567.00	6.80
60yrs	27/07/2019	0.13	0.30	23.37	0.28	573.33	6.88
5yrs	27/07/2019	0.21	0.45	34.42	0.41	676.92	8.12
Ag	27/07/2019	0.04	0.10	8.24	0.10	240.33	2.88

Table S3. Weekly rainfall amount, phosphorus concentrations and deposition in the monitoring plots.

Date	Plot	Type	Volume (mm week^{-1})	Concentration (mg P L^{-1})	Deposition (kg P week^{-1})
25/08/2018	AG1	Ag	21.50	0.24	0.02
25/08/2018	AG3	Ag	20.23	0.94	0.06
25/08/2018	AG2	Ag	48.67	0.20	0.03
25/08/2018	5Y1	5yrs	48.88	0.84	0.13
25/08/2018	5Y2	5yrs	15.42	0.61	0.03
25/08/2018	5Y3	5yrs	12.00	0.52	0.02
25/08/2018	12Y1	12yrs	14.92	2.52	0.12
25/08/2018	12Y2	12yrs	15.77	0.27	0.01
25/08/2018	12Y3	12yrs	13.79	0.86	0.04
25/08/2018	60Y1	60yrs	18.46	1.80	0.11
25/08/2018	60Y2	60yrs	22.64	1.15	0.08
25/08/2018	60Y3	60yrs	12.59	2.17	0.09
25/08/2018	20Y1	20yrs	46.16	0.18	0.03
25/08/2018	20Y2	20yrs	21.04	0.44	0.03
25/08/2018	20Y3	20yrs	11.71	0.44	0.02
1/09/2018	AG1	Ag	4.48	0.42	0.01
1/09/2018	AG3	Ag	4.95	1.77	0.03
1/09/2018	AG2	Ag	3.75	1.39	0.02
1/09/2018	5Y1	5yrs	3.71	2.11	0.03
1/09/2018	5Y2	5yrs	3.27	1.64	0.02
1/09/2018	5Y3	5yrs	4.25	1.56	0.02
1/09/2018	12Y1	12yrs	2.87	1.90	0.02
1/09/2018	12Y2	12yrs	4.24	0.36	0.00
1/09/2018	12Y3	12yrs	2.67	1.08	0.01
1/09/2018	60Y1	60yrs	4.54	1.32	0.02
1/09/2018	60Y2	60yrs	1.27	2.86	0.01
1/09/2018	60Y3	60yrs	2.99	3.19	0.03
1/09/2018	20Y1	20yrs	1.17	2.10	0.01
1/09/2018	20Y2	20yrs	4.39	1.06	0.02
1/09/2018	20Y3	20yrs	4.97	0.46	0.01
8/09/2018	AG1	Ag	65.50	0.08	0.02
8/09/2018	AG3	Ag	113.32	0.22	0.08
8/09/2018	AG2	Ag	47.25	0.12	0.02
8/09/2018	5Y1	5yrs	41.03	0.53	0.07
8/09/2018	5Y2	5yrs	90.69	0.36	0.11
8/09/2018	5Y3	5yrs	45.11	0.37	0.05
8/09/2018	12Y1	12yrs	35.76	0.44	0.05
8/09/2018	12Y2	12yrs	45.98	0.54	0.08
8/09/2018	12Y3	12yrs	89.70	0.34	0.10
8/09/2018	60Y1	60yrs	52.49	0.35	0.06
8/09/2018	60Y2	60yrs	53.48	0.50	0.09
8/09/2018	60Y3	60yrs	88.70	0.53	0.15
8/09/2018	20Y1	20yrs	51.21	0.25	0.04
8/09/2018	20Y2	20yrs	42.58	0.59	0.08
8/09/2018	20Y3	20yrs	47.82	0.23	0.04
15/09/2018	AG1	Ag	83.22	0.00	0.00
15/09/2018	AG1	Ag	83.22	0.01	0.00
15/09/2018	AG2	Ag	104.41	0.00	0.00
15/09/2018	5Y1	5yrs	100.87	0.26	0.09
15/09/2018	5Y2	5yrs	39.75	0.05	0.01
15/09/2018	5Y3	5yrs	66.49	0.35	0.08
15/09/2018	12Y1	12yrs	60.21	0.10	0.02
15/09/2018	12Y2	12yrs	142.18	0.08	0.04
15/09/2018	60Y1	60yrs	68.47	0.05	0.01
15/09/2018	60Y2	60yrs	75.72	0.13	0.03
15/09/2018	60Y3	60yrs	54.75	0.34	0.06
15/09/2018	20Y1	20yrs	91.11	0.05	0.01
15/09/2018	20Y2	20yrs	59.70	0.04	0.01
15/09/2018	20Y3	20yrs	33.11	0.08	0.01
30/09/2018	AG1	Ag	60.02	0.01	0.00
30/09/2018	AG3	Ag	57.16	0.02	0.00
30/09/2018	AG2	Ag	32.47	0.04	0.00
30/09/2018	5Y1	5yrs	33.32	0.29	0.03
30/09/2018	5Y3	5yrs	47.18	0.09	0.01
30/09/2018	12Y1	12yrs	45.05	0.09	0.01
30/09/2018	12Y2	12yrs	53.69	0.04	0.01

30/09/2018	60Y1	60yrs	50.22	0.01	0.00
30/09/2018	60Y2	60yrs	50.29	0.05	0.01
30/09/2018	60Y3	60yrs	19.28	0.80	0.05
30/09/2018	20Y2	20yrs	43.50	0.05	0.01
30/09/2018	20Y3	20yrs	31.05	0.08	0.01
20/10/2018	AG1	Ag	7.07	0.05	0.00
20/10/2018	AG2	Ag	10.72	0.15	0.01
20/10/2018	5Y3	5yrs	7.46	0.45	0.01
20/10/2018	12Y1	12yrs	4.21	0.08	0.00
20/10/2018	12Y2	12yrs	7.60	0.17	0.00
20/10/2018	60Y1	60yrs	2.97	0.96	0.01
20/10/2018	60Y2	60yrs	4.88	1.22	0.02
20/10/2018	20Y1	20yrs	9.39	0.00	0.00
20/10/2018	20Y2	20yrs	3.61	0.42	0.00
27/10/2018	AG1	Ag	80.64	0.00	0.00
27/10/2018	AG3	Ag	71.30	0.00	0.00
27/10/2018	12Y2	12yrs	50.79	0.07	0.01
27/10/2018	60Y1	60yrs	59.84	0.27	0.05
27/10/2018	60Y2	60yrs	55.88	0.16	0.03
27/10/2018	20Y1	20yrs	21.15	0.05	0.00
27/10/2018	20Y2	20yrs	69.75	0.07	0.01
3/11/2018	AG2	Ag	113.32	0.05	0.02
3/11/2018	5Y1	5yrs	98.27	0.46	0.15
3/11/2018	5Y2	5yrs	118.98	0.29	0.11
3/11/2018	60Y1	60yrs	80.36	0.18	0.05
3/11/2018	60Y2	60yrs	78.94	0.21	0.05
3/11/2018	20Y1	20yrs	94.36	0.12	0.04
3/11/2018	20Y2	20yrs	74.42	0.07	0.02
3/11/2018	20Y3	20yrs	133.84	0.15	0.07
10/11/2018	AG2	Ag	2.83	0.60	0.01
10/11/2018	5Y1	5yrs	3.25	6.27	0.07
10/11/2018	12Y3	12yrs	98.33	0.11	0.03
10/11/2018	20Y1	20yrs	1.63	2.50	0.01
17/11/2018	AG2	Ag	27.30	0.04	0.00
17/11/2018	5Y1	5yrs	24.19	0.59	0.05
17/11/2018	5Y2	5yrs	48.46	0.14	0.02
17/11/2018	5Y3	5yrs	39.33	0.11	0.01
17/11/2018	12Y1	12yrs	37.80	0.03	0.00
17/11/2018	12Y2	12yrs	44.28	0.05	0.01
17/11/2018	12Y3	12yrs	50.15	0.06	0.01
17/11/2018	60Y1	60yrs	43.93	0.11	0.02
17/11/2018	60Y2	60yrs	51.03	0.13	0.02
17/11/2018	60Y3	60yrs	62.98	0.67	0.14
17/11/2018	20Y1	20yrs	17.81	0.07	0.00
17/11/2018	20Y2	20yrs	40.32	0.09	0.01
24/11/2018	AG1	Ag	29.85	0.04	0.00
24/11/2018	AG3	Ag	28.86	0.01	0.00
24/11/2018	AG2	Ag	26.67	0.19	0.02
24/11/2018	5Y1	5yrs	25.47	0.56	0.05
24/11/2018	5Y2	5yrs	21.36	0.15	0.01
24/11/2018	5Y3	5yrs	20.80	0.31	0.02
24/11/2018	12Y1	12yrs	21.96	0.31	0.02
24/11/2018	12Y2	12yrs	25.54	0.26	0.02
24/11/2018	60Y1	60yrs	18.46	0.25	0.01
24/11/2018	60Y2	60yrs	20.58	0.07	0.00
24/11/2018	60Y3	60yrs	17.25	1.30	0.07
24/11/2018	20Y1	20yrs	31.76	0.23	0.02
24/11/2018	20Y2	20yrs	18.96	0.36	0.02
1/12/2018	AG3	Ag	1.49	1.20	0.01
1/12/2018	AG3	Ag	1.49	1.22	0.01
1/12/2018	AG2	Ag	2.55	1.55	0.01
1/12/2018	5Y1	5yrs	1.63	2.50	0.01
1/12/2018	5Y1	5yrs	1.63	3.26	0.02
1/12/2018	12Y2	12yrs	0.74	3.89	0.01
1/12/2018	60Y1	60yrs	2.33	0.99	0.01
1/12/2018	60Y3	60yrs	0.74	3.93	0.01
1/12/2018	20Y1	20yrs	2.18	1.27	0.01
1/12/2018	20Y2	20yrs	0.78	2.67	0.01
1/12/2018	20Y3	20yrs	50.57	0.34	0.06
8/12/2018	AG3	Ag	77.46	0.00	0.00
8/12/2018	AG3	Ag	77.46	0.01	0.00

8/12/2018	AG2	Ag	134.74	0.00	0.00
8/12/2018	5Y1	5yrs	146.57	0.15	0.07
8/12/2018	5Y2	5yrs	62.82	0.05	0.01
8/12/2018	5Y3	5yrs	55.60	0.00	0.00
8/12/2018	12Y1	12yrs	58.41	0.04	0.01
8/12/2018	12Y2	12yrs	77.39	0.07	0.02
8/12/2018	12Y3	12yrs	104.27	0.10	0.03
8/12/2018	60Y1	60yrs	62.53	0.04	0.01
8/12/2018	60Y2	60yrs	73.71	0.01	0.00
8/12/2018	20Y1	20yrs	112.37	0.02	0.01
8/12/2018	20Y2	20yrs	79.51	0.07	0.02
8/12/2018	20Y3	20yrs	63.52	0.02	0.00
22/12/2018	AG1	Ag	27.73	0.02	0.00
22/12/2018	5Y2	5yrs	0.81	1.35	0.00
22/12/2018	5Y3	5yrs	0.81	1.08	0.00
22/12/2018	20Y1	20yrs	4.15	0.30	0.00
22/12/2018	20Y2	20yrs	3.14	0.07	0.00
29/12/2018	AG1	Ag	5.57	0.66	0.01
29/12/2018	AG3	Ag	15.76	0.11	0.01
29/12/2018	AG2	Ag	6.56	0.20	0.00
29/12/2018	5Y1	5yrs	7.36	0.70	0.02
29/12/2018	5Y2	5yrs	3.22	0.91	0.01
29/12/2018	12Y3	12yrs	7.69	0.68	0.02
29/12/2018	12Y3	12yrs	7.69	0.35	0.01
29/12/2018	60Y1	60yrs	2.05	1.24	0.01
29/12/2018	60Y2	60yrs	2.62	1.72	0.01
29/12/2018	60Y3	60yrs	1.99	4.73	0.03
5/01/2019	AG1	Ag	53.48	0.06	0.01
5/01/2019	AG3	Ag	40.46	0.03	0.00
5/01/2019	AG2	Ag	53.62	0.05	0.01
5/01/2019	5Y1	5yrs	62.46	0.35	0.07
5/01/2019	5Y2	5yrs	17.89	0.13	0.01
5/01/2019	12Y1	12yrs	28.05	0.69	0.06
5/01/2019	12Y2	12yrs	54.68	0.17	0.03
5/01/2019	60Y2	60yrs	64.51	0.49	0.10
5/01/2019	60Y3	60yrs	62.67	0.41	0.08
5/01/2019	20Y1	20yrs	69.89	0.04	0.01
5/01/2019	20Y2	20yrs	52.73	0.36	0.06
5/01/2019	20Y3	20yrs	18.46	0.12	0.01
5/01/2019	20Y3	20yrs	18.46	0.30	0.02
12/01/2019	AG1	Ag	5.09	0.11	0.00
12/01/2019	AG3	Ag	5.23	0.14	0.00
12/01/2019	AG2	Ag	13.76	0.52	0.02
12/01/2019	5Y1	5yrs	13.79	0.51	0.02
12/01/2019	5Y2	5yrs	3.58	0.39	0.00
12/01/2019	5Y3	5yrs	25.78	0.20	0.02
12/01/2019	12Y1	12yrs	23.03	0.58	0.04
12/01/2019	12Y2	12yrs	24.05	0.17	0.01
12/01/2019	12Y3	12yrs	46.54	0.28	0.04
12/01/2019	60Y1	60yrs	1.95	1.28	0.01
12/01/2019	60Y2	60yrs	5.23	0.77	0.01
12/01/2019	60Y3	60yrs	3.14	2.19	0.02
12/01/2019	20Y1	20yrs	10.96	0.27	0.01
12/01/2019	20Y2	20yrs	2.38	1.03	0.01
12/01/2019	20Y3	20yrs	0.25	1.06	0.00
19/01/2019	AG1	Ag	21.94	0.05	0.00
19/01/2019	AG3	Ag	26.07	0.10	0.01
19/01/2019	5Y1	5yrs	14.64	2.16	0.10
19/01/2019	5Y2	5yrs	10.62	0.43	0.01
19/01/2019	5Y3	5yrs	18.59	0.73	0.04
19/01/2019	12Y1	12yrs	18.78	1.01	0.06
19/01/2019	12Y3	12yrs	10.22	0.51	0.02
19/01/2019	60Y1	60yrs	11.49	0.34	0.01
19/01/2019	60Y2	60yrs	13.55	0.40	0.02
19/01/2019	20Y1	20yrs	3.25	1.44	0.02
19/01/2019	20Y2	20yrs	12.98	0.25	0.01
26/01/2019	AG3	Ag	4.69	0.44	0.01
26/01/2019	AG2	Ag	5.80	1.49	0.03
26/01/2019	5Y1	5yrs	5.01	1.45	0.02
26/01/2019	5Y2	5yrs	3.66	0.99	0.01
26/01/2019	5Y3	5yrs	5.22	1.81	0.03

26/01/2019	12Y1	12yrs	3.75	3.66	0.04
26/01/2019	12Y2	12yrs	5.22	0.55	0.01
26/01/2019	60Y1	60yrs	4.86	1.32	0.02
26/01/2019	60Y2	60yrs	4.31	0.75	0.01
26/01/2019	60Y3	60yrs	12.31	1.66	0.07
26/01/2019	20Y2	20yrs	3.49	0.55	0.01
26/01/2019	20Y3	20yrs	18.75	0.73	0.04
2/02/2019	AG1	Ag	4.16	0.74	0.01
2/02/2019	AG3	Ag	2.87	0.36	0.00
2/02/2019	AG2	Ag	16.84	0.27	0.01
2/02/2019	5Y1	5yrs	18.11	0.59	0.04
2/02/2019	5Y2	5yrs	11.88	0.71	0.03
2/02/2019	5Y3	5yrs	3.51	1.80	0.02
2/02/2019	12Y2	12yrs	2.86	4.06	0.04
2/02/2019	12Y3	12yrs	18.04	0.39	0.02
2/02/2019	60Y1	60yrs	10.60	0.55	0.02
2/02/2019	60Y3	60yrs	27.13	0.99	0.09
2/02/2019	20Y1	20yrs	18.75	0.17	0.01
2/02/2019	20Y1	20yrs	18.75	0.17	0.01
2/02/2019	20Y2	20yrs	6.30	0.87	0.02
9/02/2019	AG1	Ag	7.31	0.46	0.01
9/02/2019	AG3	Ag	5.01	0.50	0.01
9/02/2019	AG2	Ag	7.22	0.33	0.01
9/02/2019	5Y1	5yrs	7.71	0.59	0.01
9/02/2019	5Y2	5yrs	3.88	0.91	0.01
9/02/2019	5Y3	5yrs	4.45	1.18	0.02
9/02/2019	12Y1	12yrs	3.42	1.59	0.02
9/02/2019	12Y1	12yrs	3.42	1.63	0.02
9/02/2019	12Y2	12yrs	4.77	1.18	0.02
9/02/2019	12Y3	12yrs	11.53	0.55	0.02
9/02/2019	60Y1	60yrs	4.48	2.56	0.04
9/02/2019	60Y2	60yrs	12.38	2.37	0.10
9/02/2019	20Y1	20yrs	18.75	0.30	0.02
9/02/2019	20Y3	20yrs	7.25	0.61	0.01
2/03/2019	AG1	Ag	9.05	0.27	0.01
2/03/2019	AG3	Ag	14.50	0.13	0.01
2/03/2019	AG2	Ag	31.19	0.19	0.02
2/03/2019	5Y2	5yrs	10.33	0.14	0.00
2/03/2019	5Y3	5yrs	10.75	0.47	0.02
2/03/2019	12Y1	12yrs	6.65	1.31	0.03
2/03/2019	12Y2	12yrs	9.69	0.69	0.02
2/03/2019	12Y3	12yrs	10.68	0.18	0.01
2/03/2019	60Y1	60yrs	8.84	1.40	0.04
2/03/2019	60Y2	60yrs	10.96	2.94	0.11
2/03/2019	60Y3	60yrs	9.48	3.16	0.10
2/03/2019	20Y1	20yrs	27.64	0.16	0.01
2/03/2019	20Y2	20yrs	8.86	0.53	0.02
2/03/2019	20Y3	20yrs	5.97	0.97	0.02
9/03/2019	AG3	Ag	53.48	0.04	0.01
9/03/2019	5Y1	5yrs	50.22	0.14	0.02
9/03/2019	5Y3	5yrs	34.45	0.24	0.03
9/03/2019	12Y3	12yrs	37.99	0.05	0.01
9/03/2019	60Y2	60yrs	46.83	0.42	0.06
9/03/2019	60Y3	60yrs	46.90	0.74	0.11
9/03/2019	20Y1	20yrs	56.59	0.06	0.01
9/03/2019	20Y2	20yrs	35.93	0.15	0.02
9/03/2019	20Y2	20yrs	35.93	0.08	0.01
9/03/2019	20Y3	20yrs	38.55	0.04	0.00
23/03/2019	AG1	Ag	4.01	0.29	0.00
23/03/2019	AG3	Ag	5.66	0.21	0.00
23/03/2019	AG2	Ag	18.87	0.08	0.01
23/03/2019	5Y1	5yrs	23.06	0.38	0.03
23/03/2019	5Y2	5yrs	4.87	1.18	0.02
23/03/2019	5Y3	5yrs	3.90	0.36	0.00
23/03/2019	12Y1	12yrs	3.28	0.85	0.01
23/03/2019	12Y2	12yrs	4.63	0.50	0.01
23/03/2019	12Y2	12yrs	4.63	0.23	0.00
23/03/2019	12Y2	12yrs	4.63	0.08	0.00
23/03/2019	12Y3	12yrs	6.83	0.67	0.01
23/03/2019	60Y1	60yrs	2.40	1.58	0.01
23/03/2019	60Y2	60yrs	4.07	1.88	0.02

23/03/2019	60Y3	60yrs	3.95	5.56	0.07
23/03/2019	20Y1	20yrs	8.86	0.32	0.01
23/03/2019	20Y2	20yrs	3.47	1.78	0.02
23/03/2019	20Y3	20yrs	3.93	0.68	0.01
30/03/2019	AG1	Ag	88.42	0.03	0.01
30/03/2019	AG3	Ag	104.27	0.01	0.00
30/03/2019	AG2	Ag	102.04	0.00	0.00
30/03/2019	AG2	Ag	102.04	0.00	0.00
30/03/2019	5Y1	5yrs	96.13	0.13	0.04
30/03/2019	5Y2	5yrs	76.75	0.11	0.03
30/03/2019	5Y3	5yrs	86.44	0.06	0.02
30/03/2019	12Y1	12yrs	90.09	0.07	0.02
30/03/2019	12Y2	12yrs	101.30	0.00	0.00
30/03/2019	12Y3	12yrs	78.52	0.07	0.02
30/03/2019	60Y1	60yrs	71.52	0.18	0.04
30/03/2019	60Y2	60yrs	80.50	0.15	0.04
30/03/2019	60Y3	60yrs	75.76	0.89	0.22
30/03/2019	20Y1	20yrs	86.94	0.05	0.01
30/03/2019	20Y2	20yrs	72.22	0.22	0.05
30/03/2019	20Y3	20yrs	138.15	0.05	0.02
6/04/2019	AG1	Ag	40.32	0.03	0.00
6/04/2019	AG3	Ag	50.37	0.02	0.00
6/04/2019	AG2	Ag	34.37	0.19	0.02
6/04/2019	5Y1	5yrs	35.47	0.15	0.02
6/04/2019	5Y2	5yrs	45.14	0.07	0.01
6/04/2019	12Y1	12yrs	48.21	0.16	0.03
6/04/2019	12Y2	12yrs	52.77	0.08	0.01
6/04/2019	12Y3	12yrs	38.06	0.13	0.02
6/04/2019	60Y1	60yrs	50.22	0.24	0.04
6/04/2019	60Y2	60yrs	55.53	0.38	0.07
6/04/2019	60Y3	60yrs	50.41	0.70	0.12
6/04/2019	20Y1	20yrs	38.25	0.06	0.01
6/04/2019	20Y2	20yrs	46.76	0.11	0.02
6/04/2019	20Y3	20yrs	48.79	0.13	0.02
13/04/2019	AG1	Ag	20.22	0.19	0.01
13/04/2019	AG3	Ag	22.49	0.11	0.01
13/04/2019	AG2	Ag	30.91	0.12	0.01
13/04/2019	5Y1	5yrs	32.01	1.19	0.12
13/04/2019	5Y2	5yrs	15.49	0.29	0.01
13/04/2019	5Y3	5yrs	18.39	0.41	0.02
13/04/2019	12Y1	12yrs	17.88	0.62	0.04
13/04/2019	12Y1	12yrs	17.88	0.40	0.02
13/04/2019	12Y2	12yrs	21.19	0.55	0.04
13/04/2019	12Y3	12yrs	13.65	0.32	0.01
13/04/2019	60Y1	60yrs	18.04	0.39	0.02
13/04/2019	60Y2	60yrs	18.04	0.50	0.03
13/04/2019	60Y3	60yrs	15.00	3.35	0.16
13/04/2019	20Y1	20yrs	20.23	1.42	0.09
13/04/2019	20Y3	20yrs	35.65	0.35	0.04
20/04/2019	AG1	Ag	20.87	0.10	0.01
20/04/2019	AG3	Ag	23.91	0.10	0.01
20/04/2019	AG2	Ag	11.18	0.39	0.01
20/04/2019	5Y1	5yrs	9.83	0.91	0.03
20/04/2019	5Y2	5yrs	17.00	0.26	0.01
20/04/2019	5Y2	5yrs	17.00	0.07	0.00
20/04/2019	5Y3	5yrs	17.26	0.37	0.02
20/04/2019	12Y1	12yrs	13.47	0.52	0.02
20/04/2019	12Y3	12yrs	10.61	0.55	0.02
20/04/2019	60Y1	60yrs	20.87	0.47	0.03
20/04/2019	60Y2	60yrs	18.04	0.35	0.02
20/04/2019	60Y3	60yrs	22.42	0.99	0.07
20/04/2019	20Y1	20yrs	9.90	0.60	0.02
20/04/2019	20Y2	20yrs	13.37	0.00	0.00
20/04/2019	20Y3	20yrs	10.40	0.76	0.03
27/04/2019	AG1	Ag	55.53	0.07	0.01
27/04/2019	AG3	Ag	32.11	0.04	0.00
27/04/2019	AG2	Ag	61.68	0.03	0.01
27/04/2019	5Y1	5yrs	55.46	0.26	0.05
27/04/2019	5Y2	5yrs	25.25	0.12	0.01
27/04/2019	5Y3	5yrs	25.04	0.32	0.03
27/04/2019	12Y1	12yrs	21.05	0.33	0.02

27/04/2019	12Y2	12yrs	20.94	0.06	0.00
27/04/2019	60Y1	60yrs	30.77	0.51	0.05
27/04/2019	60Y2	60yrs	27.09	0.69	0.06
27/04/2019	60Y3	60yrs	34.24	0.81	0.09
27/04/2019	20Y1	20yrs	45.84	0.10	0.01
27/04/2019	20Y2	20yrs	22.99	0.22	0.02
27/04/2019	20Y3	20yrs	32.96	0.43	0.05
4/05/2019	AG1	Ag	24.19	0.04	0.00
4/05/2019	AG3	Ag	18.38	0.12	0.01
4/05/2019	AG2	Ag	14.15	0.18	0.01
4/05/2019	5Y1	5yrs	13.94	0.65	0.03
4/05/2019	5Y3	5yrs	14.64	0.32	0.02
4/05/2019	12Y1	12yrs	13.92	0.70	0.03
4/05/2019	60Y1	60yrs	18.39	1.18	0.07
4/05/2019	60Y1	60yrs	18.39	0.03	0.00
4/05/2019	60Y2	60yrs	30.56	0.67	0.07
4/05/2019	60Y3	60yrs	15.14	2.12	0.10
4/05/2019	20Y1	20yrs	20.23	0.56	0.04
4/05/2019	20Y3	20yrs	16.98	0.73	0.04
11/05/2019	AG1	Ag	166.94	0.00	0.00
11/05/2019	AG3	Ag	146.00	0.00	0.00
11/05/2019	AG3	Ag	146.00	0.00	0.00
11/05/2019	AG2	Ag	96.49	0.00	0.00
11/05/2019	5Y1	5yrs	103.77	0.12	0.04
11/05/2019	5Y2	5yrs	121.67	0.05	0.02
11/05/2019	12Y1	12yrs	141.48	0.09	0.04
11/05/2019	12Y2	12yrs	184.48	0.08	0.05
11/05/2019	60Y1	60yrs	137.94	0.19	0.09
11/05/2019	60Y2	60yrs	152.79	0.28	0.14
11/05/2019	60Y3	60yrs	165.80	0.38	0.20
11/05/2019	20Y2	20yrs	135.11	0.04	0.02
11/05/2019	20Y3	20yrs	231.31	0.09	0.07
18/05/2019	AG1	Ag	132.92	0.01	0.01
18/05/2019	AG3	Ag	59.14	0.01	0.00
18/05/2019	AG2	Ag	70.74	0.01	0.00
18/05/2019	5Y1	5yrs	50.93	0.23	0.04
18/05/2019	5Y2	5yrs	39.54	0.14	0.02
18/05/2019	5Y3	5yrs	52.91	0.17	0.03
18/05/2019	5Y3	5yrs	52.91	0.20	0.04
18/05/2019	12Y1	12yrs	28.07	0.39	0.04
18/05/2019	12Y2	12yrs	55.46	0.14	0.03
18/05/2019	12Y3	12yrs	96.20	0.41	0.13
18/05/2019	60Y1	60yrs	44.85	1.29	0.19
18/05/2019	60Y2	60yrs	48.60	0.65	0.10
18/05/2019	60Y3	60yrs	45.56	1.49	0.22
18/05/2019	20Y1	20yrs	62.82	0.10	0.02
18/05/2019	20Y2	20yrs	37.92	0.36	0.04
25/05/2019	AG1	Ag	13.16	0.03	0.00
25/05/2019	AG3	Ag	50.93	0.05	0.01
25/05/2019	AG2	Ag	51.78	0.06	0.01
25/05/2019	5Y1	5yrs	54.04	0.22	0.04
25/05/2019	5Y3	5yrs	56.24	0.13	0.02
25/05/2019	12Y1	12yrs	50.37	0.38	0.06
25/05/2019	60Y1	60yrs	10.68	1.87	0.07
25/05/2019	60Y2	60yrs	11.74	1.78	0.07
25/05/2019	20Y1	20yrs	32.68	0.33	0.03
25/05/2019	20Y2	20yrs	10.75	0.51	0.02
25/05/2019	20Y3	20yrs	72.72	0.12	0.03
1/06/2019	AG1	Ag	38.69	0.05	0.01
1/06/2019	AG3	Ag	14.22	0.18	0.01
1/06/2019	AG2	Ag	15.14	0.09	0.00
1/06/2019	5Y1	5yrs	14.15	0.25	0.01
1/06/2019	5Y3	5yrs	15.85	0.29	0.02
1/06/2019	12Y1	12yrs	11.09	0.63	0.02
1/06/2019	12Y2	12yrs	18.11	0.45	0.03
1/06/2019	12Y3	12yrs	65.43	0.53	0.11
1/06/2019	60Y1	60yrs	29.00	1.49	0.14
1/06/2019	60Y2	60yrs	35.79	0.44	0.05
1/06/2019	60Y3	60yrs	55.18	0.74	0.13
1/06/2019	20Y1	20yrs	12.31	0.11	0.00
1/06/2019	20Y2	20yrs	24.97	0.30	0.02

1/06/2019	20Y2	20yrs	24.97	0.25	0.02
1/06/2019	20Y3	20yrs	26.74	0.13	0.01
8/06/2019	AG1	Ag	44.49	0.06	0.01
8/06/2019	AG3	Ag	23.98	0.08	0.01
8/06/2019	AG2	Ag	23.41	0.10	0.01
8/06/2019	5Y1	5yrs	22.84	0.25	0.02
8/06/2019	5Y2	5yrs	22.00	0.44	0.03
8/06/2019	5Y3	5yrs	29.99	0.21	0.02
8/06/2019	12Y1	12yrs	23.20	0.53	0.04
8/06/2019	12Y2	12yrs	34.80	0.15	0.02
8/06/2019	60Y1	60yrs	42.23	0.61	0.08
8/06/2019	60Y2	60yrs	48.74	0.44	0.07
8/06/2019	60Y3	60yrs	25.75	0.59	0.05
8/06/2019	20Y1	20yrs	38.54	0.09	0.01
8/06/2019	20Y2	20yrs	41.24	0.15	0.02
8/06/2019	20Y3	20yrs	12.18	0.63	0.02
15/06/2019	AG1	Ag	4.46	0.20	0.00
15/06/2019	AG2	Ag	7.78	0.59	0.01
15/06/2019	5Y1	5yrs	6.58	0.64	0.01
15/06/2019	12Y1	12yrs	4.87	2.64	0.04
15/06/2019	12Y3	12yrs	1.92	1.08	0.01
15/06/2019	60Y1	60yrs	4.67	0.85	0.01
15/06/2019	20Y1	20yrs	3.40	0.35	0.00
15/06/2019	20Y2	20yrs	3.47	0.73	0.01
15/06/2019	20Y3	20yrs	63.34	0.07	0.01
22/06/2019	AG1	Ag	72.01	0.04	0.01
22/06/2019	AG3	Ag	6.56	0.19	0.00
22/06/2019	AG2	Ag	52.19	0.08	0.01
22/06/2019	5Y1	5yrs	49.52	0.42	0.07
22/06/2019	5Y2	5yrs	35.16	0.20	0.02
22/06/2019	5Y3	5yrs	33.95	0.21	0.02
22/06/2019	12Y1	12yrs	32.26	0.34	0.04
22/06/2019	12Y2	12yrs	44.92	0.29	0.04
22/06/2019	12Y3	12yrs	43.50	0.26	0.04
22/06/2019	60Y1	60yrs	47.09	0.28	0.04
22/06/2019	60Y2	60yrs	56.09	0.62	0.11
22/06/2019	60Y2	60yrs	56.09	0.00	0.00
22/06/2019	60Y3	60yrs	6.15	1.32	0.03
22/06/2019	20Y1	20yrs	36.78	0.17	0.02
22/06/2019	20Y2	20yrs	46.76	0.17	0.03
22/06/2019	20Y3	20yrs	7.07	0.02	0.00
29/06/2019	AG3	Ag	40.32	0.03	0.00
29/06/2019	AG3	Ag	40.32	0.03	0.00
29/06/2019	AG2	Ag	31.62	0.04	0.00
29/06/2019	5Y1	5yrs	31.48	0.30	0.03
29/06/2019	5Y2	5yrs	33.25	0.22	0.02
29/06/2019	5Y3	5yrs	37.13	0.35	0.04
29/06/2019	12Y1	12yrs	49.46	0.15	0.02
29/06/2019	12Y2	12yrs	41.31	0.52	0.07
29/06/2019	60Y1	60yrs	18.67	0.91	0.06
29/06/2019	60Y2	60yrs	28.01	0.51	0.05
29/06/2019	60Y3	60yrs	24.40	0.65	0.05
29/06/2019	20Y1	20yrs	22.78	0.14	0.01
29/06/2019	20Y2	20yrs	25.82	0.36	0.03
29/06/2019	20Y3	20yrs	71.09	0.20	0.05
6/07/2019	AG1	Ag	12.45	0.29	0.01
6/07/2019	AG3	Ag	14.22	0.11	0.00
6/07/2019	AG2	Ag	16.71	0.10	0.01
6/07/2019	5Y1	5yrs	15.43	0.63	0.03
6/07/2019	5Y2	5yrs	12.31	0.31	0.01
6/07/2019	5Y3	5yrs	16.62	0.39	0.02
6/07/2019	5Y3	5yrs	16.62	0.34	0.02
6/07/2019	12Y1	12yrs	9.85	0.00	0.00
6/07/2019	12Y2	12yrs	13.65	0.71	0.03
6/07/2019	12Y3	12yrs	12.03	0.36	0.01
6/07/2019	60Y1	60yrs	11.67	0.01	0.00
6/07/2019	60Y1	60yrs	11.67	0.86	0.03
6/07/2019	60Y2	60yrs	11.67	1.03	0.04
6/07/2019	60Y3	60yrs	11.46	1.72	0.06
6/07/2019	20Y1	20yrs	25.61	0.36	0.03
6/07/2019	20Y2	20yrs	13.02	0.68	0.03

6/07/2019	20Y3	20yrs	85.73	0.24	0.07
13/07/2019	AG1	Ag	76.75	0.06	0.02
13/07/2019	AG3	Ag	74.98	0.03	0.01
13/07/2019	AG2	Ag	91.39	0.02	0.01
13/07/2019	5Y3	5yrs	72.93	0.26	0.06
13/07/2019	12Y1	12yrs	49.23	0.57	0.09
13/07/2019	12Y2	12yrs	65.50	0.32	0.07
13/07/2019	12Y3	12yrs	43.36	0.31	0.04
13/07/2019	60Y1	60yrs	61.54	0.64	0.13
13/07/2019	60Y2	60yrs	66.42	0.43	0.09
13/07/2019	60Y3	60yrs	68.90	0.56	0.13
13/07/2019	20Y1	20yrs	73.43	0.13	0.03
13/07/2019	20Y2	20yrs	69.32	0.16	0.04
13/07/2019	20Y3	20yrs	23.91	0.45	0.03
20/07/2019	AG1	Ag	2.47	0.65	0.01
20/07/2019	AG3	Ag	2.69	1.18	0.01
20/07/2019	AG2	Ag	2.26	0.43	0.00
20/07/2019	5Y1	5yrs	1.84	0.99	0.01
20/07/2019	5Y2	5yrs	1.67	0.89	0.00
20/07/2019	5Y3	5yrs	2.19	1.41	0.01
20/07/2019	12Y1	12yrs	0.74	3.37	0.01
20/07/2019	12Y2	12yrs	1.59	0.05	0.00
20/07/2019	12Y3	12yrs	1.70	3.85	0.02
20/07/2019	60Y1	60yrs	1.74	2.43	0.01
20/07/2019	60Y2	60yrs	1.72	2.18	0.01
20/07/2019	60Y3	60yrs	1.93	3.75	0.02
20/07/2019	20Y1	20yrs	2.19	1.10	0.01
20/07/2019	20Y2	20yrs	3.20	0.81	0.01
20/07/2019	20Y3	20yrs	2.05	1.16	0.01
27/07/2019	5Y1	5yrs	71.73	0.15	0.04
27/07/2019	5Y2	5yrs	61.82	0.24	0.05
22/09/2018	AG1	Ag	60.02	0.01	0.00
22/09/2018	AG3	Ag	57.16	0.02	0.00
22/09/2018	AG2	Ag	32.47	0.04	0.00
22/09/2018	5Y1	5yrs	33.32	0.29	0.03
22/09/2018	5Y3	5yrs	47.18	0.09	0.01
22/09/2018	12Y1	12yrs	45.05	0.09	0.01
22/09/2018	12Y2	12yrs	53.69	0.04	0.01
22/09/2018	60Y1	60yrs	50.22	0.01	0.00
22/09/2018	60Y2	60yrs	50.29	0.05	0.01
22/09/2018	60Y3	60yrs	19.28	0.80	0.05
22/09/2018	20Y2	20yrs	43.50	0.05	0.01
22/09/2018	20Y3	20yrs	31.05	0.08	0.01
13/10/2018	AG1	Ag	7.07	0.05	0.00
13/10/2018	AG2	Ag	10.72	0.15	0.01
13/10/2018	5Y3	5yrs	7.46	0.45	0.01
13/10/2018	12Y1	12yrs	4.21	0.08	0.00
13/10/2018	12Y2	12yrs	7.60	0.17	0.00
13/10/2018	60Y1	60yrs	2.97	0.96	0.01
13/10/2018	60Y2	60yrs	4.88	1.22	0.02
13/10/2018	20Y1	20yrs	9.39	0.00	0.00
13/10/2018	20Y2	20yrs	3.61	0.42	0.00

Table S4. Underlying equations and R^2 values for the relationships shown in Figure 3.

	P dep	R^2
Ag1	$P\ dep=0.0007*Volume^{0.93}$	0.13
Ag2	$P\ dep=0.4528*Volume^{-0.95}$	0.18
Ag3	$P\ dep=0.1727*Volume^{-0.83}$	0.16
5yrs1	$P\ dep=0.0152*Volume^{0.59}$	0.38
5yrs2	$P\ dep=0.007*Volume^{0.63}$	0.36
5yrs3	$P\ dep=0.0109*Volume^{0.57}$	0.10
12yrs1	$P\ dep=0.0078*Volume^{0.74}$	0.09
12yrs2	$P\ dep=0.0024*Volume^{0.9}$	0.20
12yrs3	$P\ dep=0.0077*Volume^{0.68}$	0.34
20yrs1	$P\ dep=0.0042*Volume^{0.69}$	0.04
20yrs2	$P\ dep=0.0074*Volume^{0.6}$	0.03
20yrs3	$P\ dep=0.0042*Volume^{0.78}$	0.36
60yrs1	$P\ dep=0.0068*Volume^{0.84}$	0.07
60yrs2	$P\ dep=0.0086*Volume^{0.78}$	0.00
60yrs3	$P\ dep=0.041*Volume^{0.56}$	0.70
